# Optical coherence tomography choroidal enhancement using generative deep learning

**DOI:** 10.1038/s41746-024-01119-3

**Published:** 2024-05-04

**Authors:** Valentina Bellemo, Ankit Kumar Das, Syna Sreng, Jacqueline Chua, Damon Wong, Janika Shah, Rahul Jonas, Bingyao Tan, Xinyu Liu, Xinxing Xu, Gavin Siew Wei Tan, Rupesh Agrawal, Daniel Shu Wei Ting, Liu Yong, Leopold Schmetterer

**Affiliations:** 1 Singapore Eye Research Institute, National Eye Centre, Singapore, Singapore; 2 Lee Kong Chian School of Medicine, Nanyang Technological University, Singapore, Singapore; 3 SERI-NTU Advanced Ocular Engineering (STANCE) Program, Singapore, Singapore; 4 Institute of High Performance Computing, Agency for Science, Technology and Research (A∗STAR), Singapore, Singapore; 5 Ophthalmology & Visual Sciences Academic Clinical Program (Eye ACP), Duke-NUS Medical School, Singapore, Singapore; 6 Centre for Medical Physics and Biomedical Engineering, Medical University of Vienna, Vienna, Austria; 7 University of Cologne, Faculty of Medicine and University Hospital Cologne, Department Ophthalmology, Cologne, Germany; 8 National Healthcare Group Eye Institute, Tan Tock Seng Hospital, Singapore School of Chemical and Biomedical Engineering, Nanyang Technological University (NTU), Singapore, Singapore; 9 School of Chemistry, Chemical Engineering and Biotechnology, Nanyang Technological University, Singapore, Singapore; 10 Department of Clinical Pharmacology, Medical University of Vienna, Vienna, Austria; 11 Institute of Molecular and Clinical Ophthalmology, Basel, Switzerland

**Keywords:** Eye diseases, Biomarkers

## Abstract

Spectral-domain optical coherence tomography (SDOCT) is the gold standard of imaging the eye in clinics. Penetration depth with such devices is, however, limited and visualization of the choroid, which is essential for diagnosing chorioretinal disease, remains limited. Whereas swept-source OCT (SSOCT) devices allow for visualization of the choroid these instruments are expensive and availability in praxis is limited. We present an artificial intelligence (AI)-based solution to enhance the visualization of the choroid in OCT scans and allow for quantitative measurements of choroidal metrics using generative deep learning (DL). Synthetically enhanced SDOCT B-scans with improved choroidal visibility were generated, leveraging matching images to learn deep anatomical features during the training. Using a single-center tertiary eye care institution cohort comprising a total of 362 SDOCT-SSOCT paired subjects, we trained our model with 150,784 images from 410 healthy, 192 glaucoma, and 133 diabetic retinopathy eyes. An independent external test dataset of 37,376 images from 146 eyes was deployed to assess the authenticity and quality of the synthetically enhanced SDOCT images. Experts’ ability to differentiate real versus synthetic images was poor (47.5% accuracy). Measurements of choroidal thickness, area, volume, and vascularity index, from the reference SSOCT and synthetically enhanced SDOCT, showed high Pearson’s correlations of 0.97 [95% CI: 0.96–0.98], 0.97 [0.95–0.98], 0.95 [0.92–0.98], and 0.87 [0.83–0.91], with intra-class correlation values of 0.99 [0.98–0.99], 0.98 [0.98–0.99], and 0.95 [0.96–0.98], 0.93 [0.91–0.95], respectively. Thus, our DL generative model successfully generated realistic enhanced SDOCT data that is indistinguishable from SSOCT images providing improved visualization of the choroid. This technology enabled accurate measurements of choroidal metrics previously limited by the imaging depth constraints of SDOCT. The findings open new possibilities for utilizing affordable SDOCT devices in studying the choroid in both healthy and pathological conditions.

## Introduction

The choroid, a vascular layer between the retina and sclera, plays a crucial role in maintaining ocular health^1,2^. It is associated with various retinochoroidal diseases like diabetic retinopathy (DR)^3^, age-related macular degeneration (AMD)^4^, polypoidal choroidal vasculopathy (PCV)^5^, and others^6–8^. Choroidal assessment metrics have evolved over time, from simple measurements like subfoveal thickness^9^ to more complex reconstructions like vascularity index^10^. These metrics serve as objective biomarkers for documenting choroidal status. Imaging the choroid is, however, challenging because of the anatomical location of the layer^11^.

While ultrasonography methods are limited by poor-resolution scans^12^, conventional angiography techniques with indocyanine green are limited by their invasive nature and lengthy examination time^11^. Optical coherence tomography (OCT) is the preferred clinic imaging method for non-invasive eye examination^13^, with spectral-domain OCT (SDOCT) being widely used in clinics. However, due to a progressive decrease of signal towards the choroidal structures because of scattering effects in the retinal pigment epithelium (RPE) and choriocapillaris layers, the choroidal region and the choroidal scleral interface (CSI) cannot be visualized and defined clearly. Therefore, the quantification of choroidal metrics from conventional SDOCT scans remains limited. Enhanced depth imaging (EDI) technology integrated into some SDOCT devices provides improved depth scans^14,15^, but only longer wavelengths, as in swept-source OCT (SSOCT), can fully visualize the choroid. SSOCT offers benefits like higher detection efficiency, expanded imaging range, and less sensitivity roll-off with depth^16^. Despite SSOCT’s potential in studying choroidal structures, its use is constrained to a small minority of specialized centers due to cost, resources, and expertise limitations, ultimately leading to a limited clinical appeal compared to SDOCT devices. This highlights the need to explore alternative solutions to study choroid morphology and vasculature.

The advancements in generative deep learning (DL) techniques may offer a promising avenue to enhance choroidal visualization from conventional SDOCT data. Generative DL models can artificially create new images based on real datasets and have shown potential in various applications within ophthalmology in fundus and OCT imaging^17–25^. However, the use of DL models for qualitative and quantitative assessment of retinal and choroidal biomarkers is still restricted to EDI and SSOCT scans^26–29^. Hence, the combination of SDOCT with DL may represent a cost-effective solution bridging the gap between the advantages of SSOCT and the practicality of SDOCT in clinical settings.

The objectives of this study were to (1) enhance the visualization of the choroid from conventional SDOCT scans using a generative DL model, and (2) to assess whether the choroidal metrics extracted from the synthetically enhanced SDOCT data match the measurements derived from the reference SSOCT. The motivation of this work was to improve the quality of deep eye structures from SDOCT to study processes and mechanisms of the choroid which were not assessable before in a clinical setting and opening new possibilities for utilizing affordable SDOCT devices to study the choroid in healthy and pathological conditions.

## Results

**Fig. 1:**
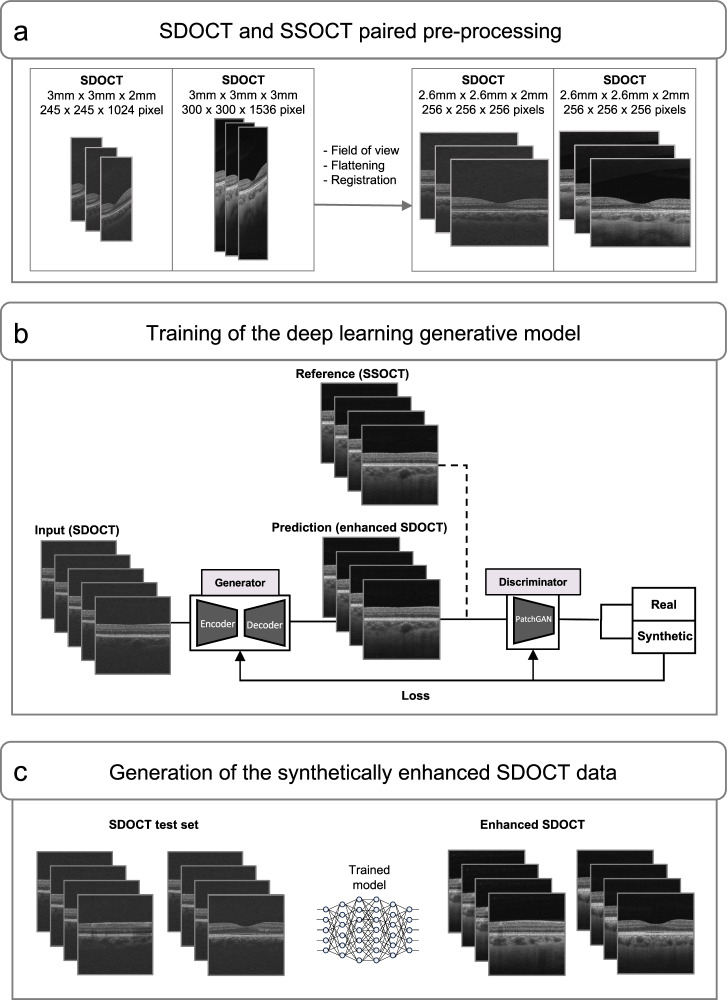
Overview of the generative deep learning approach: a pipeline for generating synthetically enhanced SDOCT enhanced data. **a** All the OCT images underwent a paired pre-processing step before being deployed in the DL model to match volume size and field of view between the two devices, align the retina, and register the image pairs. **b** During the training step, paired SDOCT and SSOCT data were used and the deep-learning model learned the deep anatomical features in the image from SSOCT scans. **c** During the testing step, SDOCT images were inputted into the trained deep learning model and synthetically enhanced SDOCT data was generated. SDOCT Spectral-Domain Optical Coherence Tomography, SSOCT Swept-Source Optical Coherence Tomography.

A generative DL model was developed and evaluated with paired SDOCT-SSOCT volumetric B-scans. After quality control and the SDOCT-SSOCT eyes pairing, a meticulous pre-processing step (Supplementary Fig. 1) was performed to match volume size and field of view between the two devices, align the retina, and accurately register the image pairs (Supplementary Fig. 2). We trained the model with a total of 589 eyes from 362 subjects using SDOCT as the input with poor choroidal visibility and matched the same 589 eyes from SSOCT as a reference for choroidal enhancement. Among the total 150,784 B-scan pairs used during the training process, 84,736 images were from healthy subjects (56.2%, 410 eyes), 39,168 data had glaucoma (26.0%, 192 eyes), and 26,880 had DR (17.8%, 133 eyes), as presented in Table 1. In the test dataset, 146 independent eyes from SDOCT, with strictly no patient overlap with the training dataset, were used to generate synthetically enhanced SDOCT data. Specifically, from a total of previously unseen 37,376 SDOCT B-scans, new 37,376 synthetically enhanced SDOCT images were generated. The same distribution of normal, glaucoma, and DR cases was maintained in the test dataset, including 20,224 images from healthy subjects (79 eyes), 9,984 from glaucoma (39 eyes), and 7,168 from DR (28 eyes) patients (Table 1). The overview of the generative DL approach is presented in Fig. 1.

**Table 1:** Study participants and data used to develop the generative deep learning model

	Train				Test				TOTAL
Data pairs	All	Normal	Glaucoma	DR	All	Normal	Glaucoma	DR	All
Subjects no.	362	202	97	63	91	51	25	15	453
Eyes no.	589	331	153	105	146	79	39	28	735
Image no.	150,784	84,736	39,168	26,880	37,376	20,224	9984	7168	188,160

The table is representative of individual SDOCT datasets and individual SSOCT datasets and shows the data pairs’ details.

*SDOCT* Spectral-Domain Optical Coherence Tomography, *SSOCT* Swept-Source Optical Coherence Tomography, *DR* Diabetic Retinopathy.

### Synthetically enhanced SDOCT image generation

**Fig. 2:**
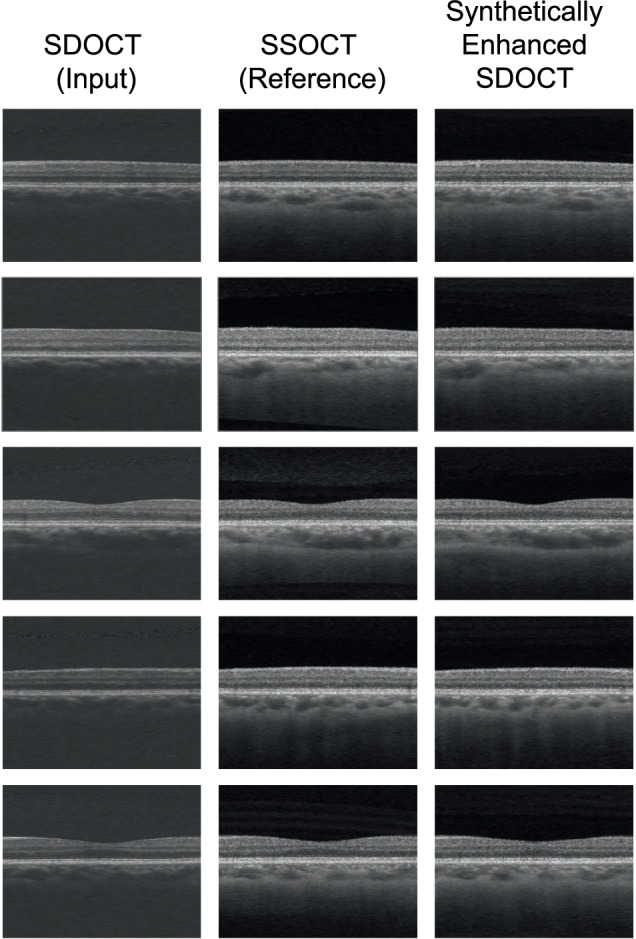
Examples of B-scans from different healthy eyes: paired SDOCT-SDOCT with their corresponding synthetically enhanced SDOCT image. Evident improvements in choroidal visualization were observed: previously obscured SDOCT deep structures became clearly visible in the synthetically enhanced SDOCT images. Since the choroidal scleral interface was visible in the synthetically enhanced SDOCT image, a visual comparison with SSOCT choroidal area could be performed, revealing a match between the vascular structures in both modalities. SDOCT Spectral-Domain Optical Coherence Tomography, SSOCT Swept-Source Optical Coherence Tomography.

**Fig. 3:**
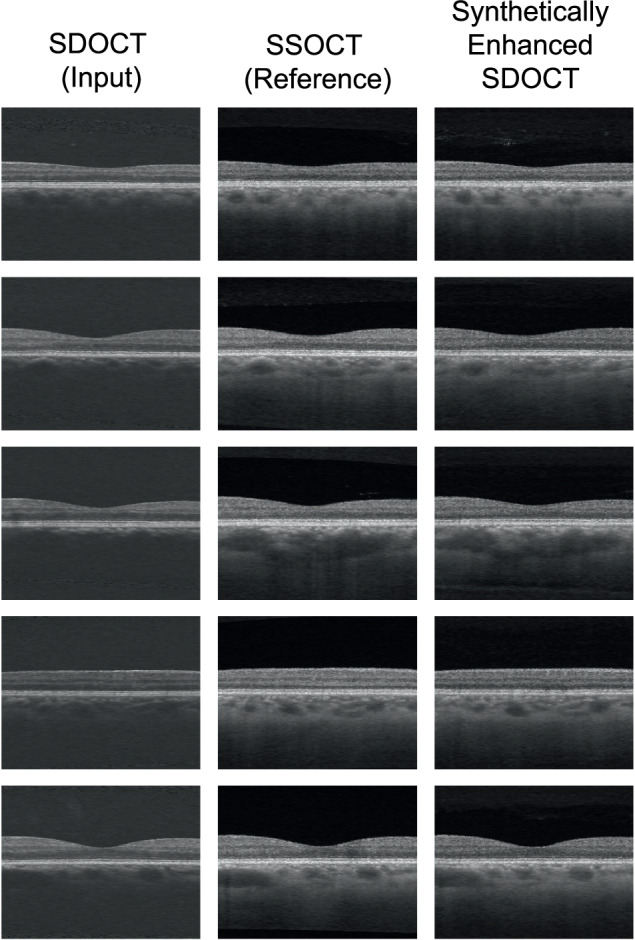
Examples of B-scans from different glaucomatous eyes: paired SDOCT-SDOCT with their corresponding synthetically enhanced SDOCT image. SDOCT Spectral-Domain Optical Coherence Tomography, SSOCT Swept-Source Optical Coherence Tomography.

**Fig. 4:**
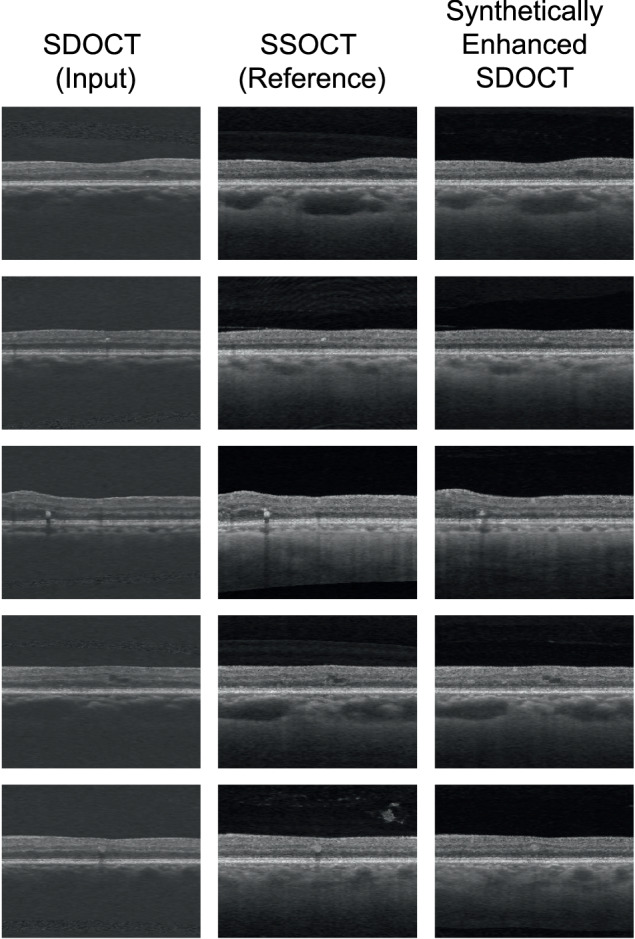
Examples of B-scans from different diabetic retinopathy eyes: paired SDOCT-SDOCT with their corresponding synthetically enhanced SDOCT image. SDOCT Spectral-Domain Optical Coherence Tomography, SSOCT Swept-Source Optical Coherence Tomography.

The SDOCT volumes in the independent test set were input in the trained generative DL model and the corresponding synthetically enhanced SDOCT images were successfully generated. The model results were evaluated for veracity via manual review of each image and 100% of the images appeared realistic to a layperson. Figures 2, 3, 4 illustrate samples of the synthetically enhanced SDOCT images in comparison with the original SDOCT and the reference SDOCT image to highlight the achieved choroidal improvement from SDOCT and choroidal structural similarity with SSOCT. From a qualitative analysis, realistic synthetic data with enhanced choroid were generated for healthy, glaucoma and DR subjects. Supplementary Fig. 3 shows a comparison of enface images from 4 different eyes at different depths to further demonstrate the improvement achieved by our DL-generated images over the input SDOCT data from a different projection.

### Experts qualitative assessment

The qualitative plausibility and authenticity of the synthetically enhanced SDOCT images were assessed by three ophthalmologists: the experts, blind from any data information, were tasked to determine whether the examined images resembled real or synthetic SSOCT scans. The expert majority correctly identified 47.5% of images as being synthetic SDOCT images or real SSOCT images, with experts 1–3’ accuracy of 45.0%, 53.0%, and 56.5%, respectively. Images from healthy subjects were correctly classified with an accuracy of 52.1%, while glaucoma and DR showed a lower clinicians’ discriminative performance of 45.5% and 46.4%, respectively. Overall sensitivities and specificities of the experts’ majority were found to be between 44.4% and 54.2%. While the less experienced ophthalmologist (expert 1) achieved the tasks with a performance below 50%, the expert with more years of experience (expert 3) performed the tasks with slightly higher accuracy of 56.5%, sensitivity of 61.2%, and specificity of 51.5%. Detailed results are presented in Table 2 and further task-specific experts’ discriminative performance is described in Supplementary Table 2. Overall, these results suggest that the clinicians could not discern between synthetically enhanced SDOCT and SSOCT.

**Table 2:** Clinicians’ assessment of the authenticity of real SDOCT and synthetically enhanced SDOCT images

	Expert Majority, %			Expert 1, %			Expert 2, %			Expert 3, %		
	Accuracy	Sensitivity	Specificity	Accuracy	Sensitivity	Specificity	Accuracy	Sensitivity	Specificity	Accuracy	Sensitivity	Specificity
All	47.5	47.6	47.4	45.0	43.7	46.4	53.0	57.3	48.5	56.5	61.2	51.5
Normal	52.1	50.0	54.2	50.0	45.8	54.2	56.2	58.3	54.2	56.2	62.5	50.0
Glaucoma	45.5	46.4	44.4	50.9	53.6	48.1	49.1	46.4	51.9	52.7	53.6	51.9
DR	46.4	47.1	45.7	39.2	37.3	41.3	53.6	62.7	43.5	58.8	64.7	52.2

The table shows the results of the ability of experts to differentiate whether the presented images were real or synthetic on visual inspection and reports the overall findings as an averaged combination of Task 1 and Task 2 outcomes. Accuracy, sensitivity, and specificity discrimination scores were calculated for a total of 30 images from normal eyes, 25 glaucoma, and 50 diabetic retinopathy eyes considering 100 images from Task 1 and 100 image pairs from Task 1. (See Supplementary Table 2 for task-specific results). *SDOCT* Spectral-Domain Optical Coherence Tomography, *SSOCT* Swept-Source Optical Coherence Tomography, *DR* Diabetic Retinopathy.

### Retinal quantitative assessment

**Fig. 5:**
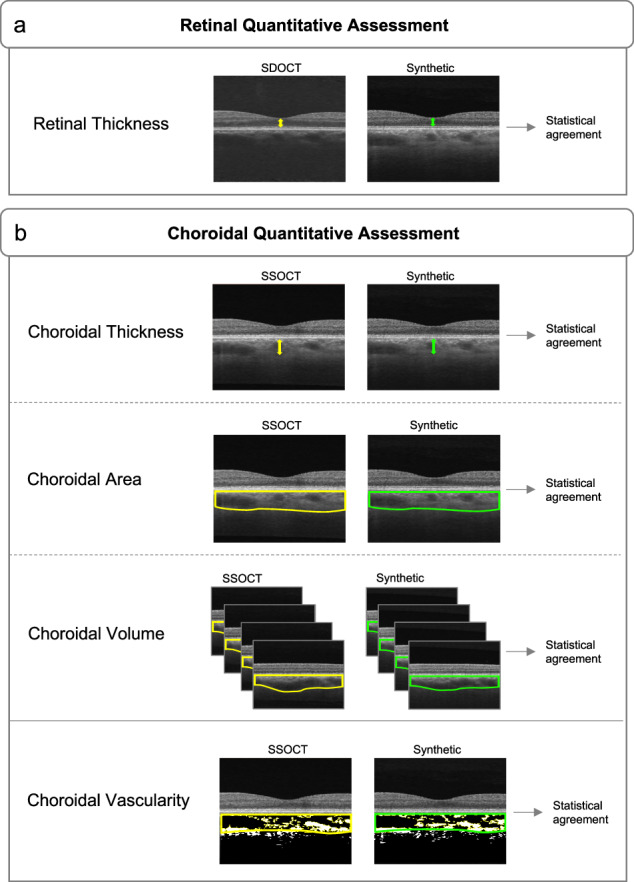
Overview of the synthetically enhanced SDOCT quantitative image evaluation approaches. **a** Retinal quantitative assessment was performed by comparing the measurements of the retinal thickness for the SDOCT data in the test set (146 eye pairs) and the corresponding synthetically enhanced SDOCT (Synthetic). **b** Choroidal quantitative assessment was performed by comparing the measurements of the choroidal morphological metrics (thickness, area, volume) and vascularity for the SSOCT data in the test set (146 eye pairs) and the corresponding synthetically enhanced SDOCT (Synthetic). SDOCT Spectral-Domain Optical Coherence Tomography, SSOCT Swept-Source Optical Coherence Tomograph.

The retinal thickness was marked from the inner limiting membrane perpendicular to the outer surface of the RPE and computed on SDOCT images and corresponding synthetically enhanced SDOCT images to assess whether the synthetic data effectively preserved the anatomical structure of the retina layers (Fig. 5a). The mean retinal thickness for both SDOCT and synthetically enhanced SDOCT images was 0.22 ± 0.02 mm (minimum of 0.18 mm and maximum of 0.26 mm) with similar values across normal, glaucoma, and DR states (Supplementary Table 3). The statistical agreement between SDOCT and synthetically enhanced SDOCT retinal thickness measurements was represented by a Pearson’s *r* of 0.95 [95% CI 0.93–0.96] and an intra-class correlation (ICC) score of 0.97 [95% CI 0.96–0.98]. All the correlations were statistically significant with *P* values < 0.001, showing significant correlations also among individual normal, glaucoma and DR states, with small mean squared errors (MAE) values between 0.003 and 0.005 (Table 3). These results indicate that synthetically enhanced SDOCT images effectively preserved the SDOCT sub-foveal retina layers.

**Table 3:** Statistical agreement results for retinal and choroidal metrics

Retinal metrics	SDOCT vs Synthetically enhanced SDOCT			
	All	Normal	Glaucoma	DR
**Retinal Thickness**				
Pearson *r* (95% CI)	0.95 *** (0.93, 0.96)	0.94 *** (0.91, 0.97)	0.96 *** (0.93, 0.98)	0.93 *** (0.84, 0.97)
ICC (95% CI)	0.97 *** (0.96, 0.98)	0.97 *** (0.96, 0.98)	0.98 *** (0.96, 0.99)	0.96 *** (0.92, 0.98)
MAE (95% CI)	0.004 (0.004, 0.005)	0.004 (0.004, 0.005)	0.003 (0.002, 0.004)	0.05 (0.004, 0.007)

Choroidal Metrics	SSOCT vs Synthetically Enhanced SDOCT			
	All	Normal	Glaucoma	DR
**Choroidal Thickness**				
Pearson r (95% CI)	0.97 *** (0.96, 0.98)	0.97 *** (0.96, 0.98)	0.96 *** (0.93, 0.98)	0.98 *** (0.97, 0.99)
ICC (95% CI)	0.99 *** (0.98, 0.99)	0.99 *** (0.98, 0.99)	0.98 *** (0.96, 0.99)	0.99 *** (0.97, 0.99)
MAE (95% CI)	0.02 (0.01, 0.02)	0.02 (0.01, 0.02)	0.02 (0.01, 0.02)	0.02 (0.01, 0.02)
**Choroidal Area**				
Pearson *r* (95% CI)	0.97 *** (0.95, 0.98)	0.95 *** (0.92, 0.97)	0.99 *** (0.98, 0.99)	0.97 *** (0.95, 0.99)
ICC (95% CI)	0.98 *** (0.98, 0.99)	0.97 *** (0.96, 0.98)	0.99 *** (0.99, 1.00)	0.99 *** (0.97, 0.99)
MAE (95% CI)	0.04 (0.03, 0.04)	0.04 (0.04, 0.05)	0.02 (0.02, 0.03)	0.03 (0.03, 0.04)
**Choroidal Volume**				
Pearson *r* (95% CI)	0.95 *** (0.92, 0.98)	0.96 *** (0.92, 0.98)	0.94 *** (0.86, 0.99)	0.95 *** (0.88, 0.99)
ICC (95% CI)	0.97 *** (0.96, 0.98)	0.98 *** (0.96, 0.98)	0.96 *** (0.92, 0.98)	0.98 *** (0.95, 0.99)
MAE (95% CI)	0.10 (0.08, 0.12)	0.10 (0.07, 0.13)	0.11 (0.06, 0.17)	0.10 (0.06, 0.14)
**Choroidal Vascularity Index**				
Pearson *r* (95% CI)	0.87 *** (0.83, 0.91)	0.86 *** (0.80, 0.90)	0.88 *** (0.77, 0.94)	0.91 *** (0.83, 0.95)
ICC (95% CI)	0.93 *** (0.91, 0.95)	0.92 *** (0.88, 0.95)	0.93 *** (0.87, 0.97)	0.95 *** (0.89, 0.98)
MAE (95% CI)	0.03 (0.03, 0.03)	0.03 (0.03, 0.04)	0.03 (0.02, 0.04)	0.02 (0.02, 0.03)

The table presents the details of the statistical correlation metrics calculated between SDOCT (input) and synthetically enhanced SDOCT data for retinal thickness and between SSOCT (reference) and synthetically enhanced SDOCT data for choroidal thickness, area, volume, and vascularity index. The analysis was conducted on the data in the test set (146 eye pairs).

*SDOCT* Spectral-Domain Optical Coherence Tomography, *SSOCT* Swept-Source Optical Coherence Tomography, *DR* Diabetic Retinopathy, *ICC* Intra-Class Correlation, *MAE* Mean Absolute Error, *CI* Confidence Interval.

*** = *P* < 0.001.

### Choroidal quantitative assessment

An overview of the synthetically enhanced quantitative choroidal image evaluation approaches is displayed in Fig. 5b. All the choroidal metrics were performed on SSOCT and corresponding synthetically enhanced SDOCT images to evaluate whether the improved generated visualization of the choroid was in agreement with the reference SSOCT. We considered choroidal thickness (CT), choroidal area (CA), and choroidal volume (CV) as morphological metrics, and the choroidal vascularity index (CVI) as vascularity metric.

**Fig. 6:**
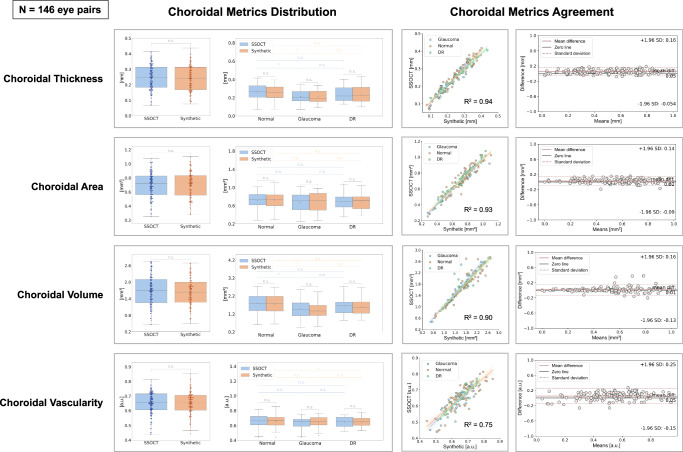
Distribution and statistical agreements of choroidal metrics between SSOCT and Synthetically Enhanced SDOCT. The boxplots illustrate the details of the distribution of choroidal metrics calculated for SSOCT and synthetically enhanced SDOCT data (Synthetic) in the test set (146 eye pairs) for choroidal thickness, area, volume, and vascularity index; *T* tests were performed to find the significant difference between the groups in a pairwise comparison. All the boxplots represent the total range of values with interquartile ranges, where the central line indicates the median and the white dot defines the median. Error bars indicate the 95% confidence intervals. The scatter plots and Bland-Altman plots show the correlation of the choroidal metrics calculated between SSOCT and DL synthetically generated SDOCT data (Synthetic) in the test set (146 eye pairs). SDOCT Spectral-Domain Optical Coherence Tomography, SSOCT Swept-Source Optical Coherence Tomography, DR Diabetic Retinopathy, n.s. not significant, *** = *P* > 0.001.; a.u. = arbitrary unit.

The choroidal region was delineated from the outer surface of the RPE to the CSI. The mean CT, CA, and CV for SSOCT and synthetically enhanced SDOCT images were 0.25 ± 0.08 mm and 0.24 ± 0.08 mm, 0.70 ± 0.18 mm^2^, and 0.71 ± 0.19 mm^2^, 1.69 ± 0.54 mm^3^ and 1.65 ± 0.51 mm^3^, with similar values across normal, glaucoma and DR states. Figure 6 and Supplementary Table 3 illustrate the details of the choroid morphological metrics measurement distributions. Overall, Pearson’s *r* and ICC scores were 0.97 [95% CI 0.96–0.98] and 0.99 [95% CI 0.98–0.99] for CT, 0.97 [95% CI 0.97–0.98] and 0.98 [95% CI 0.98–0.99] for CA, 0.95 [95% CI 0.92–0.98] and 0.97 [95% CI 0.96–0.98] for CV. All the correlations were statistically significant with *P* values < 0.001. MAE values were found to be 0.02, 0.04, and 0.10 for CT, CA, and CV, respectively. Overall significant correlations among individual normal, glaucoma and DR states were observed and are depicted in Table 3. Figure 6 further shows the morphological choroidal metrics statistical agreements from the synthetically enhanced SDOCT images with respect to the SSOCT measurements, along with scatter plots and Bland-Altman agreement plots. These results indicate that synthetically enhanced SDOCT images effectively generated clinically plausible synthetic choroids from the original SDOCT with no significant difference from SSOCT in terms of morphological choroidal metrics.

We defined the CVI as the ratio of vascular luminal area (LA) to CA and presented it as a percentage The mean CVI for SSOCT and synthetically enhanced SDOCT images was 0.65 ± 0.08 and 0.66 ± 0.08, with similar values across normal, glaucoma and DR states. Figure 6 and Supplementary Table 3 illustrate the details of the vascularity metric measurements. Overall, CVI Pearson’s *r* and ICC scores were 0.87 [95% CI 0.83–0.91] and 0.93 [95% CI 0.91–0.95]. All the correlations were statistically significant with *P* values < 0.001. MAE value for CVI was found to be 0.03, and overall significant correlations also among individual normal, glaucoma and DR states were observed (Table 3). These findings indicate that synthetically enhanced SDOCT images effectively generated plausible synthetic choroidal vasculature from the original SDOCT hidden choroidal vessels, with no significant difference from SSOCT in terms of CVI (Fig. 6).

## Discussion

The present work shows that deep structures of the posterior pole of the eye can be visualized in SDOCT images by learning from SSOCT images. This may change the way SDOCT images are used in clinics, particularly in diseases affecting the choroid. Specifically, we demonstrated (1) that the choroid visualization was significantly enhanced, as quantified by the high correlations choroidal metrics values of synthetically enhanced SDOCT data with respect to the measurements from SSOCT, and (2) that a generative DL method applied to SDOCT images can generate synthetically enhanced SDOCT data indiscernible from the reference SSOCT, as determined by the low discriminative accuracy of clinical experts. This technology improved choroidal quantitative assessments in conventional SDOCT used in clinics.

We propose the utilization of a generative DL model based on a GAN architecture^30^ to enable the majority of clinicians using SDOCT to benefit from enhanced choroidal assessment without requiring expensive and specialized equipment. Synthetically DL-generated images can be manipulated to adjust image quality and increase diversity of datasets, overcoming implications for privacy laws and data sharing^31,32^. Generative DL models have been recently deployed to reduce image variability across OCT devices, resulting in successful improvement in image quality from time-domain OCT scans to approach that of SDOCT^33^, and have contributed to reducing differences among SDOCT images derived from different vendors^34^. The DL architectures developed for these tasks are based on CycleGAN^35^, a type of GAN that allows learning a suitable translation function between the different image domains without requiring paired samples and using cycle consistency loss to help generate realistic and coherent results. However, these networks consisted of complex ensembles of CycleGANs comprising several discriminator and generator models and considering different spatial windows, eventually requiring steps of data pairing and meticulous pre-processing. Given that our dataset consisted of well-paired OCT data from two different devices, collected from the same patients during the same date of visit, we adopted a Pix2Pix GAN architecture, suitable for tasks where direct correspondences between input and output images are available. Pix2Pix was designed with a conditional GAN architecture allowing for precise control over the mapping between input and output images and is characterized by superior computational simplicity, as well as more stable training dynamics^36^. Nevertheless, there is a scarcity of studies specifically targeting the translation of SDOCT to SSOCT and the application of generative DL models to enhance choroidal visibility is an area that remained unexplored. Hence, our contribution was crucial to understanding the generative DL potential for cross-modality choroidal enhancement.

We included paired macula scans from normal subjects, as well as glaucoma and DR. Examining the choroid in normal subjects, is essential to establish baseline measurements and understand the physiological variations to recognize subtle changes that might indicate early signs of ocular diseases, allowing for timely intervention and prevention^37^. In glaucoma, studies examining the macular choroidal circulation are limited with conflicting results^38^, and allowing better visualization of macular choroidal structures may be crucial to assess the involvement in the pathogenesis of the disease, offering valuable insights into glaucoma progression and aiding in the monitoring of treatment efficacy. In DR, studying the choroid is of paramount importance as alterations in its structure and blood flow have been associated with disease severity and progression: enhanced choroidal imaging can aid in identifying early vascular changes and guide personalized therapeutic strategies to manage DR effectively^39^.

Through a visual comparison of SDOCT and DL-generated images, evident improvements in choroidal visualization were observed: previously obscured structures due to increased noise levels become clearly visible in the synthetically enhanced SDOCT images from both cross-sectional (Figs. 2, 3, and 4) and enface (Supplementary Fig. 3) projections. Since the CSI was visible in the synthetically enhanced SDOCT image, a visual comparison with SSOCT choroidal area could be performed, revealing a match between the vascular structures in both modalities. To assess the qualitative plausibility and authenticity of the synthetically enhanced SDOCT images, a subset of SSOCT and synthetically enhanced SDOCT data were presented for blind evaluation to three clinicians. The subset comprises 50% of DR data to emphasize the evaluation of images where the disease exhibits more pronounced manifestations. Sensitivities and specificities of clinical expert 3 (senior consultant) were higher in the task involving the grading based on pairs of real-synthetic images in the DR subset (Supplementary Table 2). Generally, clinicians could not accurately discriminate the synthetically enhanced SDOCT images from the real data (Table 2). Therefore, the synthetically generated SSOCT images closely resembled the SSOCT images.

Through a quantitative analysis, we determined that retinal structure was not compromised: with a Pearson’s *r* of 0.95 and an ICC score of 0.97 with respect to the original SDOCT, we demonstrated that the DL model generated images with effectively preserved retinal thickness (Table 3). Through further quantitative assessment of the synthetically generated SSOCT images, we found that the choroidal metrics values match the high correlation of the choroidal metrics with the SSOCT data. Choroidal metrics are objective biomarkers that can facilitate reproducible and reliable measurements, allowing the documentation of the effects of ageing on the choroid as well as pathological processes and response to pharmacological intervention. Several studies have proposed CT and CA as markers to assess disease conditions^37^ reporting them as indicators of ocular^9,40,41^ and systemic health^42–47^. We obtained a strong significant agreement between synthetically enhanced SDOCT and SSOCT measurements from Pearson’s *r* of 0.97 and ICC up to 0.99: this indicates that the DL model generated images with effectively enhanced choroidal thickness and area (Fig. 6, Table 3). Similar findings were discovered when assessing the choroidal morphology from a volumetric perspective. The DL model used for CV calculation was previously trained on PlexElite scans of non-flattened images: some outliers in the correlation plots were expected given the diversity of our images set (Fig. 6). Nevertheless, excellent agreement performance was reached (Table 3, Fig. 6).

On the other hand, CVI allows vascular analyses of the choroid^10,37,48,49^ and has been assessed in studies on retinitis pigmentosa^50^, central serous chorioretinopathy (CSC)^51,52^, branch retinal vein occlusion^53^, retinal dystrophies^54^, and Alzheimer’s disease^55^. We analyzed CVI to gain further insights beyond choroidal morphology and assess the proportion of vascular LA within the choroid. Our study enabled precise CVI measurements from synthetically enhanced SDOCT data and demonstrated that they exhibited no significant difference compared to measurements from SSOCT. These measurements, which were not attainable from the original SDOCT data, showed a high Pearson’s *r* of 0.87 and an ICC score of 0.93, highlighting the reliability and consistency of our approach (Table 3, Fig. 6). Additionally, novel automated techniques to enhance the efficiency of CVI estimation, facilitating a faster calculation process yet providing accurate measurements, will be explored^56,57^.

In this study, we present an approach to translate SDOCT images into choroid-enhanced images using a state-of-the-art DL image synthesis technique, trained on a diverse dataset including normal, glaucoma, and DR cases. While SDOCT data is readily available in clinics, SSOCT scans are not routinely performed and are only available to few specialized referral centers. Our proposed methodology yielded robust and flexible results, as evidenced by the strong statistical agreements of choroidal metrics. Although training a generative DL model may pose computational challenges, this limitation becomes negligible in practice since the algorithm generated synthetically enhanced SDOCT data offline. Our study was constrained by the availability of OCT scans from only two modalities, Cirrus and PlexElite, obtained as paired data in the clinical study. The scarcity of paired data involving different modalities and OCT devices from different manufacturers limits the opportunity to investigate improvements in OCT scans from one modality based on data from another. As a result, investigations focusing on enhancing OCT scans using data from different modalities during the same visit remain uncommon in the existing literature. Strategies for data collection and collaboration between different clinical centers would be crucial to gather an adequate dataset for training and validation. Collaborative efforts would facilitate the creation of a larger and more diverse database, improving the DL model’s accuracy and applicability.

Future work will aim to extend our approach to generate enhanced choroidal data from eyes with various other diseases that impact the choroid, such as CSC, PCV, and retinal vascular diseases. Additionally, we will focus on studying choroidal changes and exploring how our synthetically enhanced SDOCT data can improve the assessment of these changes compared to standard clinical data. By including a broader range of ocular pathologies, we can assess the generalizability and robustness of our synthetic enhancement technique in diverse clinical scenarios. Beyond ophthalmology, our DL approach could enhance OCT image quality across various medical fields, such as dermatology, where high-resolution imaging is pivotal for diagnosing skin conditions. Extending the potential of our image-to-image methodology to diverse medical imaging modalities and applications, including multimodal imaging settings, could contribute to the broader landscape of medical DL research and facilitate advancements in diagnostic imaging and patient care. For instance, in magnetic resonance imaging, where scan quality and contrast are crucial for accurate diagnosis, our framework could generate enhanced images and aid in the detection and characterization of abnormalities from conventional machines operating at low magnetic field strengths.

To ease the requirements for paired data and preserve the fine disease feature details when considering scans from eyes with chorioretinal conditions, the integration of diffusion models^58^ in the enhanced image generation process will be explored. Furthermore, unsupervised domain adaptation^59^ recent advances may also offer an opportunity to overcome generalizability limitations when dealing with unseen or out-of-distribution data^60^. We will focus on the adoption of our approach in real-world scenarios, considering rigorous validation and standardization to enhance the credibility and acceptance of the synthetic data in clinical decision-making, providing user-friendly interfaces and a computationally efficient solution.

## Methods

This study included data from normal, glaucoma, and DR participants derived from a clinical study performed at the Singapore Eye Research Institute in Singapore, a single-center tertiary eye care institution. All studies complied with the tenets of the Declaration of Helsinki and were approved by the SingHealth Centralized Institutional Review Board. Written informed consent was obtained from all the participants.

### Study participants

Study participants were enrolled from the Singapore Imaging Eye Network, a clinical cross-sectional study, and collected between 2018 and 2021 from Chinese, Malay, and Indian patients aged above 21 years. Supplementary Table 1 describes the demographics of the study participants. Normal eyes were defined as individuals free from clinically relevant eye conditions such as glaucoma/-suspect/self-report glaucoma^61,62^ and retinopathies^63^. Glaucomatous eyes were defined as having a pathological optic disc appearance with a corresponding glaucoma hemifield test outside normal limits; primary open-angle glaucoma, primary angle-closure glaucoma, normal tension glaucoma, and ocular hypertension were included^62^. DR was defined as present if any characteristic lesion as defined by the Early Treatment Diabetic Retinopathy Study severity scale was found^64^. Patients with co-diagnosis of clinically relevant eye diseases such as retinal diseases and AMD^65^, or significant media opacity, were excluded from recruitment as they might interfere with the aim of the study. We excluded participants for poor image quality and incomplete or missing data. We finally included a total of 453 patients (253 healthy participants, 122 glaucoma patients, and 78 diabetic retinopathy patients).

### OCT imaging—spectral-domain OCT

The OCT imaging was first performed using an SDOCT (Cirrus 5000, Carl Zeiss Meditec, Dublin, California, USA) characterized by a light source wavelength of 840 nm, scanning speed of 68,000 A-scan/s, axial and transverse resolution in tissue of 5 μm and 15 μm respectively. Each SDOCT data volume consisted of 245 A-scans and 245 B-scans with a field of view of 3 mm × 3 mm × 2 mm.

### OCT imaging—swept-source OCT

Subsequently, with a time gap in the order of minutes, the second imaging was conducted using a prototype SSOCT system (PlexElite 9000, Zeiss Meditec, Dublin, California, USA) with a central wavelength scanning laser of 1,050 nm, system operation speed of 100,000 to 200,000 A-scan/s, and axial and lateral resolutions in tissue of 6.3 µm and 20 µm respectivelyEach SSOCT data volume consists of 300 A-scans and 300 B-scans covering a 3 mm x 3 mm x 3 mm volume.

### OCT data pairing

A review software (Zeiss Meditec, Dublin, California, USA) was used to extract the B-scans. We included in our study paired OCT data acquired by deploying a 3 mm x 3 mm scanning protocol centered at the fovea. The proprietary signal strength extracted from the two OCT devices, documented using a scale from 1 to 10, was utilized as a quality index for preliminary data inclusion/exclusion. Only OCT with signal strength 6 and above were considered according to the manufacturer’s recommendation. Subsequently, one trained ophthalmic technician scanned all the participants and manually reviewed the quality of the scans. Scans were excluded from further analysis if one or more of the following criteria were met: poor clarity images, weak or inconsistent signal intensity across the scan caused by obstacles, and excessive motion artifacts. The SDOCT-SSOCT image pairing was performed at eye level. If the SDOCT and SSOCT scans for both patient eyes met the eligibility criteria above mentioned, both eyes were included in the study; if only one patient eye met the eligibility criteria, only the eligible eye was included in the study.

### Retinal thickness

The thickness of the retina was manually measured at the center of the fovea. To determine the foveal pit, we scanned through several B-scans to establish the thinnest retina layer, and the neighboring two B-scans were averaged for the segmentation of the choroidal-scleral boundary^66^. The subfoveal retinal thickness was marked from the inner limiting membrane perpendicular to the outer surface of the RPE. The thickness, measured with a customized caliper function (MATLAB R2020b, MathWorks, MA, USA) by an OCT expert (V.B.), was presented in a pixel unit and subsequently converted to millimeters via axial digital sampling to match the scanning field of view.

### Choroidal thickness

The thickness of the choroid was manually measured at the center of the fovea, determining the foveal pit with the procedure previously described for retinal thickness^66^. The CT was perpendicularly marked from the outer surface of the RPE to the CSI and manually measured with the same function utilized for retinal thickness by the OCT expert (V.B.). Similarly, CT was converted to millimeters via axial digital sampling.

### Choroidal area

The area of the choroid was computed from the central foveal OCT scan of each volume after image brightness and contrast adjustment to allow a more precise selection of CA boundaries, using Fiji software (Image J 1.54b; http://imagej.nih.gov/ij/). To calculate CA, we used the RPE-Bruch’s membrane estimation derived from the pre-processing step and manually delineated the CSI using a polygon tool. The pixel area was converted to millimeters^2^ via axial and transverse digital sampling to match the scanning field of view. This step was manually performed to ensure precise CA segmentation, as it is directly followed by the CVI estimation.

### Choroidal volume

The volume of the choroid was automatically calculated for each eye. The 3D CV measure was performed with a DL model^67^ which utilizes a U-Net-based architecture^68^ fused with a multi-task learning approach to segment the choroid from three-dimensional OCT aggregating the spatial context from adjacent cross-sectional slices. The resulting pixel volume was converted to millimeters^3^ via volumetric digital sampling to match the scanning field of view.

### Choroidal vascularity index

The vascularity index was computed using Fiji software after the segmentation of the CA from the central foveal OCT scan of each volume. We defined CVI as the ratio of vascular LA to CA and presented it as a percentage^37^. To calculate LA, image binarization was performed using Niblack’s auto-local thresholding technique^69,70^, and a color threshold tool was applied to automatically delineate the LA (dark pixels). Finally, CVI was computed by dividing LA by CA^10,37^.

### OCT images paired pre-processing

After the SDOCT-SSOCT eyes pairing step, the images underwent pre-processing to be deployed in the generative DL model (Fig. 1a). The detailed data pre-processing framework can be found in Supplementary Fig. 1. First, cropping and 3D Lanczos interpolations^71,72^ were performed to match volume size and field of view between SDOCT and SSOCT scans. Lanczos interpolation technique uses a sinc function as a convolution kernel to achieve high-quality resampling while minimizing aliasing artifacts and preserving fine details. Second, a de-speckling algorithm based on anisotropic diffusion filtering was applied to enhance the quality of the scans and an automated RPE location estimation was used to flatten the retina and align the corresponding OCT pairs. Third, a customized intensity-based enface registration was performed for each SDOCT-SSOCT pair to further fine-align the field of view. In Supplementary Fig. 2 we describe the registration accuracy analysis. A further step of cropping and field of view adjustments was applied to obtain the final dataset. Each OCT eye pair finally consisted of 256 scans per volume and 256×256 pixels images, covering a field of view of 2.6 mm × 2.6 mm × 2 mm.

### Generative DL model development

Out of the 735 eyes satisfying the inclusion criteria, we used 589 eyes for the training of the DL model (150,784 images) and 146 independent eyes to test the DL model (37,376 images) and generate the synthetically enhanced SDOCT data. There was strictly no overlap with patients between the train and test set. We used a generative DL model based on Pix2Pix architecture^30^ for image synthesis to automatically translate SDOCT scans to choroidal-enhanced images based on SSOCT. The model learned deep anatomical features from SSOCT and applied choroid properties to SDOCT images. During the training process, SDOCT and SSOCT images were loaded pairwise into the model. Pix2Pix is a generative adversarial network (GAN) designed for image-to-image translation and requires paired and well-pixel-wise aligned images. Briefly, the DL architecture is comprised of a generator model which takes as input SDOCT images and creates new plausible choroidal-enhanced SDOCT images, and a discriminator model that classifies images as real (SSOCT) or fake (synthetic), to determine whether the synthetic data is acceptable transformation of the SSOCT image (Fig. 1b). The two models are trained simultaneously in an adversarial process where the generator seeks to better fool the discriminator and the discriminator tries to better identify the counterfeit images^30^. Specifically, the generator used in this work is a Resnet-9 architecture which makes use of residual connections^73^ and is composed of three encoding blocks, nine residual blocks and three decoding blocks. Each encoding block consists of a convolution, followed by instance norm and Relu layers and each of the residual blocks follows the convolution-InstanceNorm-ReLU-Dropout-convolution-InstanceNorm residual connection structure. The discriminator consists of 5 layers and uses a patch-wise method that only penalizes structure at the scale of patches. While most complex discriminators in GAN architectures utilize the whole image for establishing a synthetic or real (0 or 1) value, our PatchGAN tries to classify if each N × N patch in an image is real or synthetic with the advantage to be applied to arbitrarily large images, utilize fewer training parameters, and run faster. In this work, we use a patch size of 70 $\times$ 70. Supplementary Fig. 4. shows the diagrams of the generator and discriminator with the details of each block. One single generative DL model was trained for normal, glaucoma, and DR. data. The development of the generative DL model was done in Python (Python Software Foundation, DE, USA) and trained for approximately 24 hours and 15 epochs using a GeForce RTX 2080 Ti GPU; Adam optimizer with a learning rate of −0.0001 was set. After training was completed, an independent SDOCT dataset consisting of the same disease distribution as the training dataset was used to generate the synthetically enhanced SDOCT data (Fig. 1c).

### Clinician image evaluation

We evaluated the qualitative plausibility and authenticity of the synthetically enhanced SDOCT images providing a subset of SSOCT and corresponding synthetically enhanced SDOCT data to 3 ophthalmologists for visual evaluation and manual grading (Expert 1: R.J., ophthalmologist resident; Expert 2: J.S., senior staff registrar with >5 years of experience in ophthalmology; Expert 3: G.S.W.T, senior retina consultant with >15 years of experience in ophthalmology). First, from the 146 eye pairs in the independent test set, a total of 100 randomly selected single images (50 SSOCT and 50 synthetically enhanced SDOCT images) were presented to the clinicians. The experts were asked to report whether they believed each image was real or synthetic. Second, a total of 100 randomly selected SSOCT-synthetic image pairs were inspected: the clinical experts were asked to report which of the 2 images in each pair was real (SSOCT). For the two tasks, a total of 30 images from normal eyes, 25 glaucoma, and 50 DR were used. Expert majority predictions and overall task predictions for all images were also calculated. Individual experts’ predictions and the experts’ majority predictions were compared with the ground truth. The images were prepared on a digital grading form and the clinicians were allowed to review the images at a setting and time of their convenience. No prior information regarding the data distribution was given to avoid any bias.

### Retinal and choroidal metrics evaluation

Retinal and choroidal metrics were computed for the independent test set data and extracted as mentioned in a previous section. Retinal thickness measurements were manually performed on SDOCT images and corresponding synthetically enhanced SDOCT images to assess whether the synthetic data effectively preserved the anatomical structure of the retina layers; statistical agreement and error between the two sets of measurements were subsequently computed. All the choroidal metrics were performed on SSOCT and corresponding synthetically enhanced SDOCT images to evaluate whether the improved generated visualization of the choroid was in agreement with the reference SSOCT. Statistical agreement and error between the two sets of CT, CA, CV, and CVI measurements were subsequently computed. Figure 5 shows how the retinal and choroidal metrics are extracted respectively from SDOCT, SSOCT and the corresponding synthetic data.

### Statistical analysis

Evaluation metrics for clinical experts’ ability to discern between synthetic and real SSOCT images were accuracy, sensitivity, and specificity. We quantified the retinal thickness preservation of the synthetically enhanced SDOCT data over SDOCT images and the choroidal enhancement of the synthetically enhanced SDOCT data over SSOCT images by comparing the agreement of CT, CA, CV, and CVI with scatter plots and Bland-Altman plots. We computed Pearson’s correlation coefficients (*r*), ICC scores, and MAE values to quantify the agreement with real OCT and synthetically enhanced SDOCT data. Confidence interval values of 95% were generated using bootstrap (5000 iterations) and *P* values were calculated using *F*-test with scores less than 0.001 considered statistically significant. All the statistical analysis was done using Python and the scikit-learn library.

### Reporting summary

Further information on research design is available in the Nature Research Reporting Summary linked to this article.

### Supplementary information

Supplementary material

Reporting Summary

## Data Availability

The de-identified patient data and any data that support the findings of this study may be shared on reasonable request to the corresponding author, subject to approval from the SingHealth Centralised Institutional Review Board.

## Code availability

Any custom code used to implement the deep learning model detailed in this paper and all data analysis scripts will be made available upon reasonable request to the corresponding author and establishment of data sharing agreement between institutions.
