# Supplementary material for: Optical coherence tomography choroidal enhancement using generative deep learning
Source: NPJ Digit Med. 2024 May 4;7:115. doi: 10.1038/s41746-024-01119-3 (PMC11069520; doi:10.1038/s41746-024-01119-3)
Supplement: Supplementary file 1 — Supplementary material [file 41746_2024_1119_MOESM1_ESM.pdf]

Supplementary Material

Supplementary Figure 1. **Data pre-processing.**

i. **Field of view matching**

In our data extraction process from SDOCT and SSOCT, we obtained OCT data using a standardized 3mm x 3mm scanning protocol. However, each OCT machine yielded B-scans with varying field of view and digital resolution. Compatibility and consistency of the OCT data is critical for the subsequent analyses. Specifically, SDOCT captured images covering 3mm x 3mm x 2mm volumes with B-scans sized at 245 x 1024 pixel and 245 scans per volume. On the other hand, SSOCT covered 3mm x 3mm x 3mm areas with B-scans at 300 x 1536 pixel and 300 scans per volume. Therefore, we first addressed these discrepancies ensuring uniform magnification and same scanning volumes across both devices.

a. **Cropping**

To match the field of view between SDOCT (3mm x 3mm x 2mm) and SSOCT (3mm x 3mm x 3mm) raw eye volumes, a cropping step was performed on SSOCT data to reduce the z dimension and obtain equivalent transverse magnification.

b. **Interpolation**

To pair the digital resolution between SDOCT (245 x 245 x 1024 pixel) with SSOCT (300 x 300 x 1024 pixel) eye volumes, Lanczos interpolation[1,2] technique was applied and matching 256 x 256 x 1024 pixel scans were obtained. Lanczos interpolation involved multiplying the original data by a windowing sinc function. The windowed data was then convolved with a 3D sinc kernel, the weighted sum at each position was calculated, and finally, the result was normalized. The size of the neighborhood considered during the interpolation process was adjusted to achieve high-quality resampling while minimizing aliasing artifacts. Fine retinal and choroidal features in both up-sampling (SDOCT) and down-sampling (SSOCT) processes were preserved. The enface digital resolution of 256 x 256 pixel was applied to fit the Dl model input size requirements.

ii. **Flattening**

To optimize the training of our generative DL model, the SDOCT-SSOCT image pairs alignment is crucial. OCT scans of the same eye obtained from different machines

during the same visit can exhibit variations in orientation or y-offsets. Therefore, we perform data alignment after flattening OCT layers.

a. De-speckling

OCT images inherently exhibit speckle noise. An anisotropic diffusion filtering[3,4] to effectively denoise the images diminishing the presence of speckles was applied to all the images. In anisotropic diffusion filtering, the diffusion process varies in different directions across the image along the edges and contours to smooth out noise while preserving the structural details. The diffusion process is controlled by a diffusion coefficient that depends on the local gradient of the image. The de-specking was applied to enhance the OCT image contrast and facilitate the subsequent step of retinal pigment epithelium (RPE) segmentation.

b. Automatic RPE estimation

The segmentation of the RPE layer was based on pixel intensity: for each column (A-line) of the B-scans, the brightest pixel was automatically assigned as a preliminary estimate of the RPE. From this initial estimation, we automatically identified and removed discontinuities greater than 30 pixels (often associated with background artifacts or retinal nerve fiber layer) and pixels lying in columns that presented a signal-to-noise ratio significantly low. Finally, the RPE was delineated fitting the remaining points with a second-order polynomial.

c. Retina and choroid alignment

The RPE line was horizontally straightened and positioned in the middle of the y-axis for all the B-scans. To force the RPE points to lie in the flat line, each column (A-line) of the B-scans was shifted, up or down, with a fixed y-axis offset guided by the polynomial RPE fit. This step ensured retinal and choroidal flattening with preliminary alignment of SDOCT-SSOCT data pairs.

iii. Registration

The OCT data pairs included in this study are characterized by a macula-centered 3mm x 3mm protocol, however, the potential impact of operator variability, patient breathing, and other involuntary movements during the scanning procedures must be considered. Therefore, to achieve precise alignment of OCT pairs and ensure more accurate matching of the field of view, we incorporated an image registration step. Effective image registration relies on the automatic detection and alignment of prominent features within the images. The choroidal layer features, characterized by

small and often indistinct blood vessels, do not serve as optimal candidates for this purpose. Additionally, in SDOCT data the larger vasculature of the deep choroid is not clearly visible. Therefore, we have opted for a registration based on the superficial retinal vessels, as they represent distinctive and reliable features conducive to an effective registration process.

a. Enface projections

Enface OCT images were obtained by extracting the x-y plane projections from the volumetric B-scans, therefore creating 1024 slices for each eye volume. The retina region was automatically identified and the minimum projection of 20 retina enface scans per volume pair was generated to enhance retinal vessel contrast in the image.

b. Rigid transformations

These augmented retina enface scans were used to perform the automated intensity-based rigid registration, which consisted of a map calculation of 2D (x,z) pixel translations and rotations. Assuming that for each eye volume the shifts calculated in the retina were consistent with the translations in the below choroidal region, we propagated the registration maps to the whole SDOCT enface volumes.

c. Final adjustments

We aligned SDOCT enface volumes to match SSOCT data obtaining SDOCT images with void areas corresponding to the translation and rotation shifts. To eliminate these regions, a final field of view cropping was performed to SDOCT and SSOCT enface volumes. The final field of view of the paired OCT volumes consisted of 2.6mm x 2.6mm x 2mm. The B-scans were then reconstructed extracting the x-z plane projections from the registered enface volumes. A final step of Lanczos interpolation was performed to obtain final B-scans of 256 x 256 pixel digital resolution, required as DL model input.

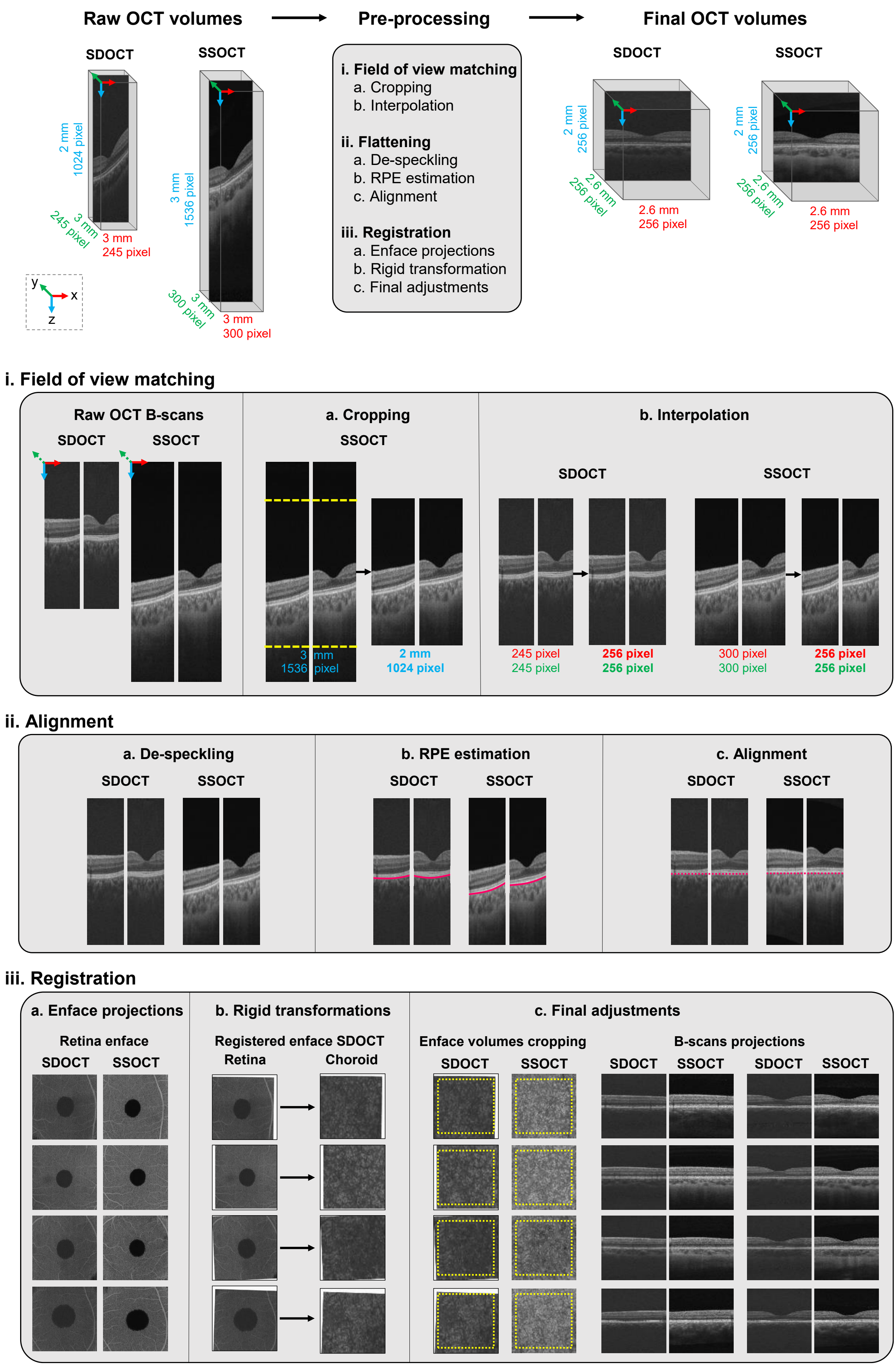

SDOCT= Spectral-Domain Optical Coherence Tomography; SSOCT= Swept-Source Optical Coherence Tomography.

<u>Supplementary Figure 2.</u> Data pre-processing: registration accuracy. Examples of the enface image registration from four different eyes. The registration accuracy is demonstrated by displaying qualitative analysis (top) of the overlay SDOCT and registered SDOCT with the SSOCT enface retina images, and quantitative analysis performed computing error and similarity metrics (bottom), proving that the pre-processing method generated well-aligned registered data pairs. Error bars indicate the 95% confidence intervals.

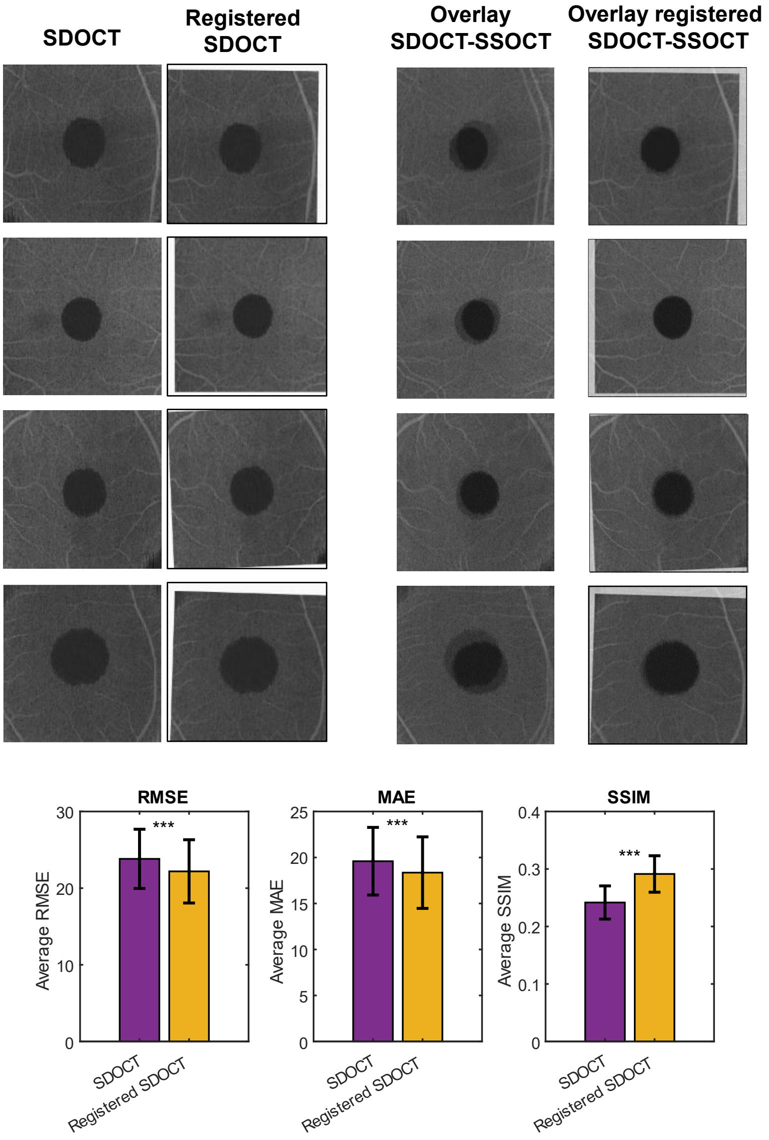

SDOCT= Spectral-Domain Optical Coherence Tomography; SSOCT= Swept-Source Optical Coherence Tomography.; RMSE = Root Mean Squared Error; Mean Absolute Error; SSIM = Structural Similarity Index Metric; *** = P<0.001.

Supplementary Figure 3. Enface results. Comparison of enface images from four different eyes: SDOCT (left), SSOCT (center), and synthetically enhanced SDOCT choroidal (right) enface scans at different depths and corresponding cross-sectional image (top). We further demonstrate, with enface projections, that our synthetically enhanced SDOCT choroidal scans improve the input SDOCT images providing choroidal vasculature details previously hidden by the high noise level.

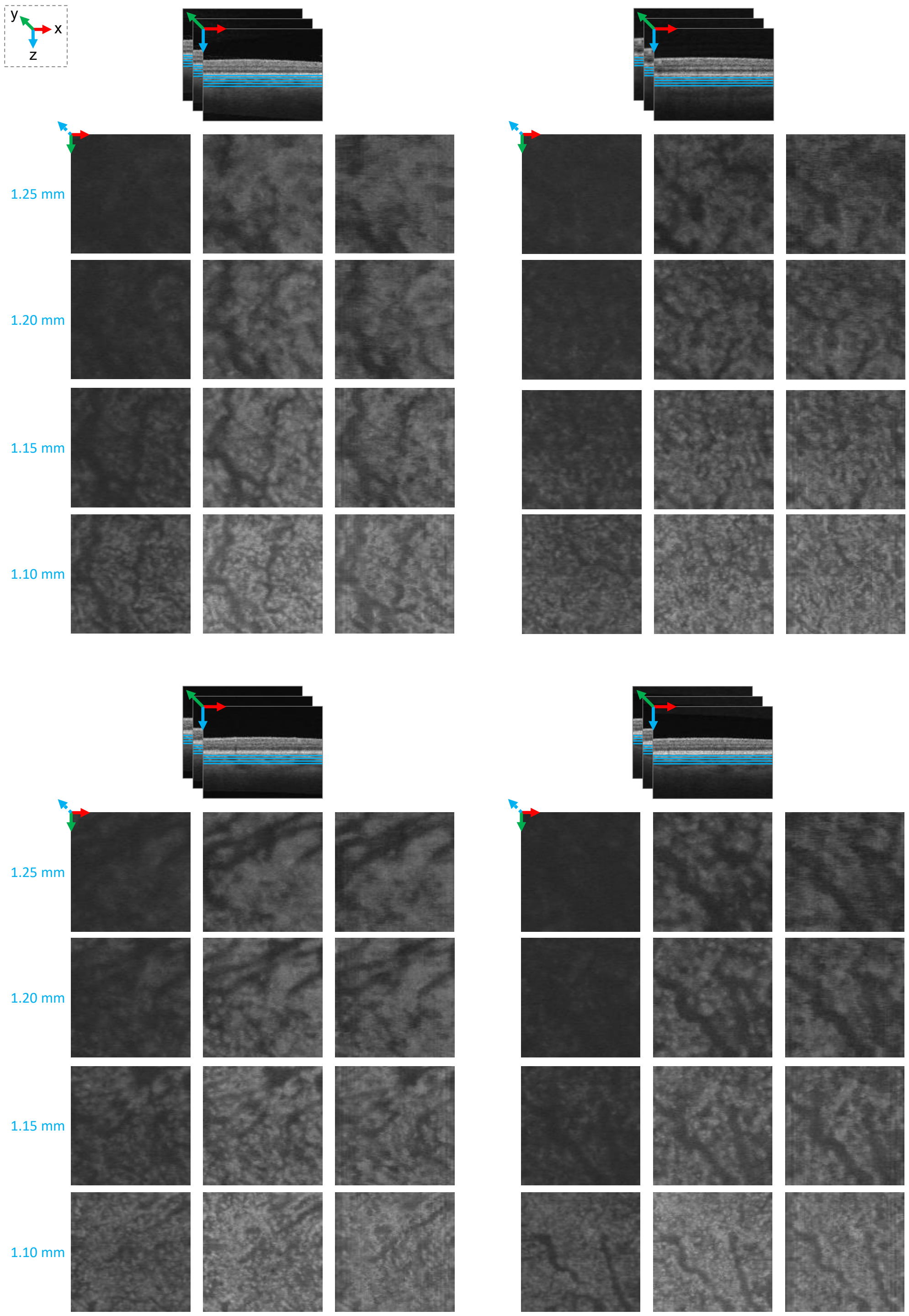

<u>Supplementary Figure 4.</u> Deep learning model architecture: generator and discriminator networks.

Generator Network

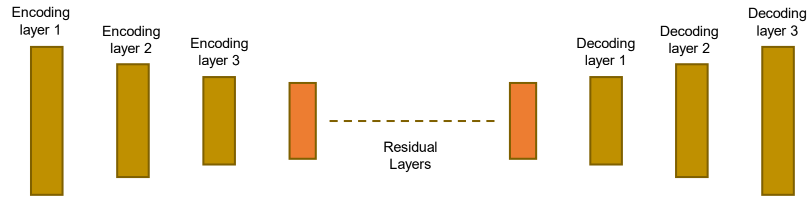

Block	Layer
Encoding Block 1	64 × 7 × 7 conv, stride 1<br>Instance norm<br>Relu
Encoding Block 2	128 × 3 × 3 conv, stride 2<br>Instance norm<br>Relu
Encoding Block 3	256 × 3 × 3 conv, stride 2<br>Instance norm<br>Relu

Block	Layer
Residual block 1	256 × 3 × 3 conv, stride 1<br>Instance norm<br>Relu<br>Dropout(p=0.5)<br>256 × 3 × 3 conv, stride 1<br>Instance norm
Residual block 2	256 × 3 × 3 conv, stride 1<br>Instance norm<br>Relu<br>Dropout(p=0.5)<br>256 × 3 × 3 conv, stride 1<br>Instance norm
Residual block 3	256 × 3 × 3 conv, stride 1<br>Instance norm<br>Relu<br>Dropout(p=0.5)<br>256 × 3 × 3 conv, stride 1<br>Instance norm
Residual block 4	256 × 3 × 3 conv, stride 1<br>Instance norm<br>Relu<br>Dropout(p=0.5)<br>256 × 3 × 3 conv, stride 1<br>Instance norm
Residual block 5	256 × 3 × 3 conv, stride 1<br>Instance norm<br>Relu<br>Dropout(p=0.5)<br>256 × 3 × 3 conv, stride 1<br>Instance norm
Residual block 6	256 × 3 × 3 conv, stride 1<br>Instance norm<br>Relu<br>Dropout(p=0.5)<br>256 × 3 × 3 conv, stride 1<br>Instance norm
Residual block 7	256 × 3 × 3 conv, stride 1<br>Instance norm<br>Relu<br>Dropout(p=0.5)<br>256 × 3 × 3 conv, stride 1<br>Instance norm
Residual block 8	256 × 3 × 3 conv, stride 1<br>Instance norm<br>Relu<br>Dropout(p=0.5)<br>256 × 3 × 3 conv, stride 1<br>Instance norm
Residual block 9	256 × 3 × 3 conv, stride 1<br>Instance norm<br>Relu<br>Dropout(p=0.5)<br>256 × 3 × 3 conv, stride 1<br>Instance norm

Block	Layer
Decoding Block 1	128 × 3 × 3 conv, stride 2<br>Instance norm<br>Relu
Decoding Block 2	64 × 3 × 3 conv, stride 2<br>Instance norm<br>Relu
Decoding Block 3	64 × 3 × 3 conv, stride 2<br>Instance norm<br>Tanh

4

Discriminator Network

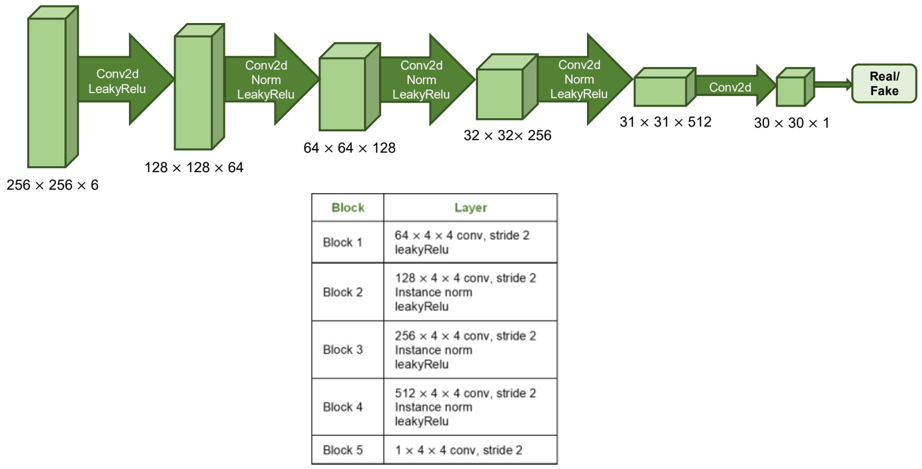

Block	Layer
Block 1	64 × 4 × 4 conv, stride 2<br>leakyRelu
Block 2	128 × 4 × 4 conv, stride 2<br>Instance norm<br>leakyRelu
Block 3	256 × 4 × 4 conv, stride 2<br>Instance norm<br>leakyRelu
Block 4	512 × 4 × 4 conv, stride 2<br>Instance norm<br>leakyRelu
Block 5	1 × 4 × 4 conv, stride 2

References

1. Lanczos C. A precision approximation of the gamma function. Journal of the Society for Industrial and Applied Mathematics, Series B: Numerical Analysis. 1964;1(1):86-96.
2. Moraes T, Amorim P, Da Silva JV, Pedrini H. Medical image interpolation based on 3D Lanczos filtering. Computer Methods in Biomechanics and Biomedical Engineering: Imaging & Visualization. 2020;8(3):294-300.
3. Perona P, Malik J. Scale-space and edge detection using anisotropic diffusion. IEEE Transactions on pattern analysis and machine intelligence. 1990;12(7):629-639.
4. Gerig G, Kubler O, Kikinis R, Jolesz FA. Nonlinear anisotropic filtering of MRI data. IEEE Transactions on medical imaging. 1992;11(2):221-232.

<u>Supplementary Table 1.</u> Summary of the patient demographics.

	Train				Test				TOTAL
	**All**	**Normal**	**Glaucoma**	**DR**	**All**	**Normal**	**Glaucoma**	**DR**	**All**
Gender, Female (%)	176 (48.6)	112 (55.4)	43 (44.7)	21 (33.3)	47 (51.6)	31 (60.8)	9 (36.0)	7 (46.7)	223 (49.2)
Age, mean ± SD	53.9 ± 14.7	54.5 ± 14.8	55.0 ± 14.2	51.3 ± 14.2	54.1 ± 14.6	53.6 ± 14.7	62.8 ± 10.5	51.7 ± 14.3	54.0 ± 14.6
Refractive Error (D)	-1.5 ± 3.7	-1.6 ± 3.7	-1.4 ± 3.7	-1.6 ± 2.6	-1.5 ± 3.7	-1.6 ± 3.7	-1.1 ± 5.5	-1.6 ± 2.7	-1.5 ± 3.7
Signal Strength, mean ± SD									
SDOCT (Cirrus)	8.8 ± 1.3	8.8 ± 1.3	8.7 ± 1.3	8.7 ± 1.3	8.8 ± 1.3	8.8 ± 1.3	8.8 ± 1.3	8.7 ± 1.4	8.8 ± 1.3
SSOCT (PlexElite)	9.1 ± 0.8	9.1 ± 0.8	9.0 ± 0.8	9.1 ± 0.6	9.0 ± 0.8	9.0 ± 0.8	8.6 ± 0.9	9.1 ± 0.7	9.0 ± 0.8

D = Diopters; SDOCT = Spectral-Domain Optical Coherence Tomography; SSOCT = Swept-Source Optical Coherence Tomography; DR = Diabetic Retinopathy; SD = Standard Deviation.

<u>Supplementary Table 2.</u> Clinicians' discrimination performance: results of the assessment of the authenticity of real SSOCT and synthetically enhanced SDOCT images from Task 1 and Task 2. The ability of experts to differentiate whether the presented images were real or synthetic on visual inspection is reported. Task 1: clinicians were provided with synthetically enhanced SDOCT and SSOCT images and asked to differentiate whether each of the images they visually inspected was real or synthetic; accuracy, sensitivity, and specificity scores of discrimination were calculated from a total of 100 images (50 real and 50 synthetic, blinded distribution). Task 2: clinicians were provided with pairs of synthetically enhanced SDOCT and SSOCT images and asked to indicate which of the 2 images in the pair was real; accuracy, sensitivity, and specificity scores of discrimination were calculated for 100 image pairs. For the two tasks, a total of 30 images from normal eyes, 25 glaucoma, and 50 DR were used.

	Expert Majority, %			Expert 1, %			Expert 2, %			Expert 3, %		
	**Accuracy**	**Sensitivity**	**Specificity**	**Accuracy**	**Sensitivity**	**Specificity**	**Accuracy**	**Sensitivity**	**Specificity**	**Accuracy**	**Sensitivity**	**Specificity**
**Task 1,** n = 100												
All	56.0	66.0	50.0	55.0	54.0	56.0	55.0	68.0	42.0	53.0	54.0	52.0
Normal	50.0	55.6	44.4	44.4	33.3	55.6	50.0	77.8	22.2	66.7	66.7	66.7
Glaucoma	66.7	73.3	60.0	70.0	73.3	66.7	60.7	66.7	53.3	53.3	53.3	53.3
DR	51.9	57.7	46.2	50.0	50.0	50.0	53.8	65.4	42.3	48.1	50.0	46.2
**Task 2,** n = 100 pairs												
All	39.0	34.0	44.7	35.0	34.0	36.2	51.0	47.2	55.3	60.0	67.9	51.1
Normal	53.3	46.7	60.0	55.3	55.3	55.3	60.0	46.7	73.3	50.0	60.0	40.0
Glaucoma	20.0	15.4	25.0	28.0	30.8	25.0	36.0	23.1	50.0	52.0	53.8	50.0
DR	40.0	36.0	45.0	26.7	24.0	30.0	53.3	60.0	45.0	71.1	80.0	60.0

SDOCT= Spectral-Domain Optical Coherence Tomography; SSOCT= Swept-Source Optical Coherence Tomography; DR = Diabetic Retinopathy.

<u>Supplementary Table 3.</u> Retinal and choroidal metrics measurements. The table presents the details of the retinal thickness calculated between SDOCT (input) and synthetically enhanced SDOCT data and the details of choroidal thickness, area, volume, and vascularity index calculated between SSOCT (reference) and synthetically enhanced SDOCT. The analysis was conducted on the data in the test set (146 eye pairs).

Retinal Metrics	**SDOCT**				**Synthetically Enhanced SDOCT**			
	All	Normal	Glaucoma	DR	All	Normal	Glaucoma	DR
**Retinal Thickness**								
mean ± SD [mm]	0.22 ± 0.02	0.22 ± 0.02	0.22 ± 0.02	0.21 ± 0.02	0.22 ± 0.02	0.22 ± 0.02	0.22 ± 0.02	0.22 ± 0.02
min [mm]	0.18	0.18	0.18	0.18	0.18	0.18	0.18	0.18
max [mm]	0.26	0.26	0.26	0.25	0.26	0.26	0.25	0.25

Choroidal Metrics	**SSOCT**				**Synthetically Enhanced SDOCT**			
	All	Normal	Glaucoma	DR	All	Normal	Glaucoma	DR
**Choroidal Thickness**								
mean ± SD [mm]	0.25 ± 0.08	0.26 ± 0.08	0.21 ± 0.07	0.24 ± 0.08	0.24 ± 0.08	0.26 ± 0.08	0.21 ± 0.07	0.24 ± 0.09
min [mm]	0.07	0.07	0.71	0.13	0.08	0.08	0.09	0.10
max [mm]	0.42	0.42	0.35	0.41	0.44	0.40	0.34	0.44
**Choroidal Area**								
mean ± SD [mm^2]	0.70 ± 0.18	0.72 ± 0.17	0.67 ± 0.20	0.69 ± 0.17	0.71 ± 0.19	0.73 ± 0.18	0.67 ± 0.21	0.69 ± 0.18
min [mm^2]	0.25	0.28	0.25	0.35	0.28	0.29	0.28	0.37
max [mm^2]	1.08	1.02	1.03	1.08	1.11	1.11	0.98	1.04
**Choroidal Volume**								
mean ± SD [mm^3]	1.69 ± 0.54	1.79 ± 0.53	1.49 ± 0.55	1.65 ± 0.47	1.65 ± 0.51	1.77 ± 0.51	1.41 ± 0.47	1.61 ± 0.45
min [mm^3]	0.47	0.60	0.47	0.86	0.51	0.65	0.51	0.86

max [mm^3]	2.75	2.75	2.58	2.74	2.70	2.70	2.43	2.67
**Choroidal Vascularity Index**								
mean ± SD [a.u]	0.65 ± 0.08	0.66 ± 0.08	0.63 ± 0.08	0.65 ± 0.07	0.66 ± 0.08	0.66 ± 0.08	0.64 ± 0.08	0.65 ± 0.07
min [a.u,]	0.44	0.44	0.44	0.52	0.44	0.44	0.46	0.53
max [a.u.]	0.81	0.81	0.75	0.74	0.85	0.85	0.76	0.77

SDOCT= Spectral-Domain Optical Coherence Tomography; SSOCT= Swept-Source Optical Coherence Tomography; DR= Diabetic Retinopathy; SD= Standard Deviation; a.u. = arbitrary
