# Supplementary material for: Optical coherence tomography choroidal enhancement using generative deep learning
Source: NPJ Digit Med. 2024 May 4;7:115. doi: 10.1038/s41746-024-01119-3 (PMC11069520; doi:10.1038/s41746-024-01119-3)
Supplement: Supplementary file 2 — Reporting Summary [file 41746_2024_1119_MOESM2_ESM.pdf]

nature portfolio

Corresponding author(s): Prof Leopold Schmetterer

Last updated by author(s): Jan 30, 2024

Reporting Summary

Nature Portfolio wishes to improve the reproducibility of the work that we publish. This form provides structure for consistency and transparency in reporting. For further information on Nature Portfolio policies, see our Editorial Policies and the Editorial Policy Checklist.

Statistics

For all statistical analyses, confirm that the following items are present in the figure legend, table legend, main text, or Methods section.

n/a	Confirmed	
☐	☒	The exact sample size (*n*) for each experimental group/condition, given as a discrete number and unit of measurement
☐	☒	A statement on whether measurements were taken from distinct samples or whether the same sample was measured repeatedly
☐	☒	The statistical test(s) used AND whether they are one- or two-sided<br>*Only common tests should be described solely by name; describe more complex techniques in the Methods section.*
☒	☐	A description of all covariates tested
☒	☐	A description of any assumptions or corrections, such as tests of normality and adjustment for multiple comparisons
☐	☒	A full description of the statistical parameters including central tendency (e.g. means) or other basic estimates (e.g. regression coefficient) AND variation (e.g. standard deviation) or associated estimates of uncertainty (e.g. confidence intervals)
☐	☒	For null hypothesis testing, the test statistic (e.g. *F*, *t*, *r*) with confidence intervals, effect sizes, degrees of freedom and *P* value noted<br>*Give P values as exact values whenever suitable.*
☒	☐	For Bayesian analysis, information on the choice of priors and Markov chain Monte Carlo settings
☒	☐	For hierarchical and complex designs, identification of the appropriate level for tests and full reporting of outcomes
☐	☒	Estimates of effect sizes (e.g. Cohen's *d*, Pearson's *r*), indicating how they were calculated

Our web collection on statistics for biologists contains articles on many of the points above.

Software and code

Policy information about availability of computer code

Data collection	Data collection software used in this study were provided by OCT equipment manufacturers: Cirrus 5000 v11.0.029946 and PlexElite 9000 v2.1.0.55513 (Carl Zeiss Meditec, CA, USA).
Data analysis	The data pre-processing and the choroidal metrics estimations were performed in MATLAB R2020b (MathWorks, MA, USA) and Image J 1.54b (http://imagej.nih.gov/ij/). The deep learning model was developed and deployed using standard model libraries and scripts in PyTorch 1.9 and all the statistical analysis was performed using scikit-learn library 1.1.2 (Python 3.9, DE, USA).

For manuscripts utilizing custom algorithms or software that are central to the research but not yet described in published literature, software must be made available to editors and reviewers. We strongly encourage code deposition in a community repository (e.g. GitHub). See the Nature Portfolio guidelines for submitting code & software for further information.

Data

Policy information about availability of data

All manuscripts must include a data availability statement. This statement should provide the following information, where applicable:

- Accession codes, unique identifiers, or web links for publicly available datasets
- A description of any restrictions on data availability
- For clinical datasets or third party data, please ensure that the statement adheres to our policy

The de-identified patient data and any data that support the findings of this study may be shared on reasonable request to the corresponding author, subject to approval from the SingHealth Centralised Institutional Review Board.

Research involving human participants, their data, or biological material

Policy information about studies with human participants or human data. See also policy information about sex, gender (identity/presentation), and sexual orientation and race, ethnicity and racism.

Reporting on sex and gender	Supplementary Table 1 reports the gender composition of the study population (50.8% male; 49.2% female) as extracted from the electronic health record.
Reporting on race, ethnicity, or other socially relevant groupings	Study participants were enrolled from a clinical study conducted in Singapore, resulting in an ethnicity composition predominantly comprising Chinese, Malay, and Indian patients.
Population characteristics	Study participants were enrolled from the Singapore Imaging Eye Network, a clinical cross-sectional study performed at the Singapore Eye Research Institute in Singapore, a single-center tertiary eye care institution, and collected between 2018 and 2021 from patients aged above 21 years. Table 1 and Supplementary Table 1 reports the study population characteristics.
Recruitment	This study included included adult patients with the ability to provide written informed consent. We included paired OCT data acquired from two OCT devices deploying a 3mm×3mm scanning protocol centered at the fovea with signal strength ≥ 6 from healthy subjects and patients with glaucoma and diabetic retinopathy. We excluded patients with co-diagnosis of clinically relevant eye diseases, poor image quality and incomplete or missing data.
Ethics oversight	All procedures performed were in adherence with the ethical standards of the SingHealth Centralized Institutional Review Board (CIRB Ref No. 2018/2020). Written informed consent was obtained from all participants in accordance with the Declaration of Helsinki.

Note that full information on the approval of the study protocol must also be provided in the manuscript.

Field-specific reporting

Please select the one below that is the best fit for your research. If you are not sure, read the appropriate sections before making your selection.

☒ Life sciences ☐ Behavioural & social sciences ☐ Ecological, evolutionary & environmental sciences

For a reference copy of the document with all sections, see nature.com/documents/nr-reporting-summary-flat.pdf

Life sciences study design

All studies must disclose on these points even when the disclosure is negative.

Sample size	Sample size was not explicitly calculated as we utilized all the paired data comprising both SDOCT and SSOCT eye scans available in the Singapore Imaging Eye Network clinical study. With a total of 188,160 pairs of images from 735 eyes of 453 subjects, the substantial dataset size ensured robust model development and testing.
Data exclusions	The OCT image pairing was performed at eye level: we excluded patients with both eyes not meeting the eligibility criteria. If only one patient eye met the eligibility criteria, only the not eligible eye was excluded from the study.
Replication	Experiment findings were replicated with our provided code scripts. Confidence interval values of 95% were generated using bootstrap with 5000 replicates.
Randomization	The images used to develop the deep learning model were randomly partitioned into training and testing data sets. The subset of paired images provided to the clinicians for authenticity assessment was randomly selected from the test data set.
Blinding	The clinicians were blinded for the distribution of real and synthetic data during the authenticity assessment. The OCT expert who performed the manual choroidal metrics measurements was blinded for the inspection of real or synthetic data to avoid any bias.

Reporting for specific materials, systems and methods

We require information from authors about some types of materials, experimental systems and methods used in many studies. Here, indicate whether each material, system or method listed is relevant to your study. If you are not sure if a list item applies to your research, read the appropriate section before selecting a response.

Materials & experimental systems

n/a	Involved in the study
☒	☐ Antibodies
☒	☐ Eukaryotic cell lines
☒	☐ Palaeontology and archaeology
☒	☐ Animals and other organisms
☐	☒ Clinical data
☒	☐ Dual use research of concern
☒	☐ Plants

Methods

n/a	Involved in the study
☒	☐ ChIP-seq
☒	☐ Flow cytometry
☒	☐ MRI-based neuroimaging

Clinical data

Policy information about clinical studies

All manuscripts should comply with the ICMJE guidelines for publication of clinical research and a completed CONSORT checklist must be included with all submissions.

Clinical trial registration	N/A, retrospective study
Study protocol	SIENA: Singapore Imaging Eye Network, R1500/83/2017
Data collection	Retrospective. This study included data from normal, glaucoma, and diabetic retinopathy participants derived from a clinical study performed at the Singapore Eye Research Institute in Singapore, a single-center tertiary eye care institution. Study participants were enrolled from the Singapore Imaging Eye Network, a clinical cross-sectional study, and data were collected between 2018 and 2021.
Outcomes	Synthetically enhanced SDOCT images from the deep learning model. Accuracy, sensitivity, and specificity from the clinicians' authenticity assessment of the synthetic data. Pearson's r, intra class correlation, mean absolute error from retinal thickness of synthetic data versus SDOCT and choroidal thickness, area, volume, vascularity index of synthetic data versus SSOCT.

Plants

Seed stocks	N/A
Novel plant genotypes	N/A
Authentication	N/A
